# {2,2′-[*o*-Phenyl­enebis(nitrilo­methyl­idyne)]diphenolato}dipyridinecobalt(III) perchlorate

**DOI:** 10.1107/S1600536809027330

**Published:** 2009-07-18

**Authors:** Mehdi Salehi, Soraia Meghdadi, Mehdi Amirnasr, Kurt Mereiter

**Affiliations:** a Department of Chemistry, Isfahan University of Technology, Isfahan 84156-83111, Iran; b Faculty of Chemistry, Vienna University of Technology, Getreidemarkt 9/164SC, A-1060 Vienna, Austria

## Abstract

The title compound, [Co(C_20_H_14_N_2_O_2_)(C_5_H_5_N)_2_]ClO_4_ or [Co(salophen)(py)_2_]ClO_4_, where salophen is *o*-phenyl­enebis(nitrilo­methyl­idyne)]diphenolate and py is pyridine, contains a six-coordinate mononuclear cobalt(III) atom. The two phenolic O atoms and the two imine N atoms are located in *cis* positions. There are two pyridine mol­ecules attached to the metal atom, filling the axial sites with a mutually perpendicular disposition of the pyridine planes [86.11 (5)°]. The Co complexes are stacked in layers parallel to (100). Coherence of the structure is provided by a variety of C—H⋯O interactions between the complexes and the perchlor­ate counter anion.

## Related literature

For general background to transition metal Schiff-base complexes with a tetra­dentate N_2_O_2_ ligand configuration, see: Schenk *et al.* (2007 ▶); Yamada (1999 ▶). For related Co complexes, see: Amirnasr *et al.* (2001 ▶); Khandar *et al.* (2007 ▶). For oxygenation and oxidation reactions of related Co complexes, see: Nishinaga & Tomita (1980 ▶); Park *et al.* (1998 ▶); Speiser & Stahl (1995 ▶). For the anti­microbial activity of related Co complexes, see: Kumar *et al.* (2009 ▶); Miodragović *et al.* (2006 ▶); Mishra *et al.* (2008 ▶).
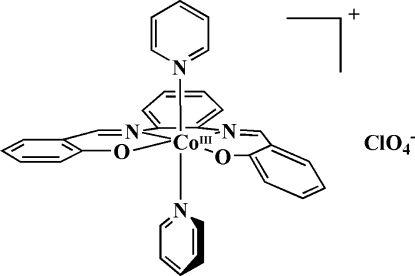


## Experimental

### 

#### Crystal data

[Co(C_20_H_14_N_2_O_2_)(C_5_H_5_N)_2_]ClO_4_
*M* *_r_* = 630.91
Monoclinic, 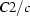

*a* = 33.4032 (16) Å
*b* = 10.6586 (5) Å
*c* = 16.3498 (8) Å
β = 112.179 (1)°
*V* = 5390.3 (4) Å^3^
*Z* = 8
Mo *K*α radiation
μ = 0.79 mm^−1^
*T* = 200 K
0.44 × 0.18 × 0.07 mm

#### Data collection

Bruker APEXII CCD diffractometer
Absorption correction: multi-scan (*SADABS*; Bruker, 2008 ▶) *T*_min_ = 0.86, *T*_max_ = 0.95
24638 measured reflections
7804 independent reflections
6268 reflections with *I* > 2σ(*I*)
*R*_int_ = 0.024

#### Refinement

*R*[*F* ^2^ > 2σ(*F* ^2^)] = 0.031
*wR*(*F* ^2^) = 0.086
*S* = 1.03
7804 reflections
379 parameters
H-atom parameters constrained
Δρ_max_ = 0.38 e Å^−3^
Δρ_min_ = −0.40 e Å^−3^

### 

Data collection: *APEX2* (Bruker, 2008 ▶); cell refinement: *SAINT* (Bruker, 2008 ▶); data reduction: *SAINT*; program(s) used to solve structure: *SHELXS97* (Sheldrick, 2008 ▶); program(s) used to refine structure: *SHELXL97* (Sheldrick, 2008 ▶); molecular graphics: *SHELXTL* (Sheldrick, 2008 ▶); software used to prepare material for publication: *SHELXTL*.

## Supplementary Material

Crystal structure: contains datablocks I, global. DOI: 10.1107/S1600536809027330/dn2470sup1.cif

Structure factors: contains datablocks I. DOI: 10.1107/S1600536809027330/dn2470Isup2.hkl

Additional supplementary materials:  crystallographic information; 3D view; checkCIF report

## Figures and Tables

**Table 1:** Hydrogen-bond geometry (Å, °)

*D*—H⋯*A*	*D*—H	H⋯*A*	*D*⋯*A*	*D*—H⋯*A*
C5—H5⋯O4^i^	0.95	2.40	3.293 (3)	157
C11—H11⋯O6^ii^	0.95	2.49	3.261 (3)	138
C12—H12⋯O3	0.95	2.59	3.501 (2)	161
C14—H14⋯O3^iii^	0.95	2.51	3.002 (2)	112
C29—H29⋯O1^iv^	0.95	2.47	3.111 (2)	124

Symmetry codes: (i) 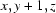; (ii) 
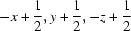
; (iii) 
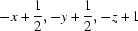
; (iv) 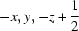.

**Fig. 1.**
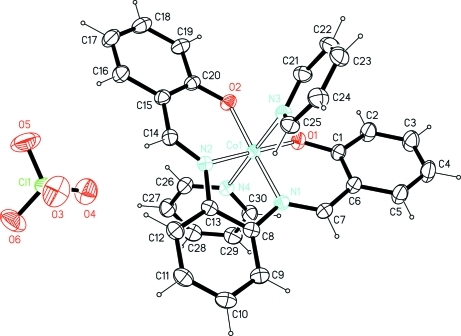
The ORTEP drawing of (I), with the atom labeling scheme. Displacement ellipsoids are drawn at the 50% probability level.

**Fig. 2.**
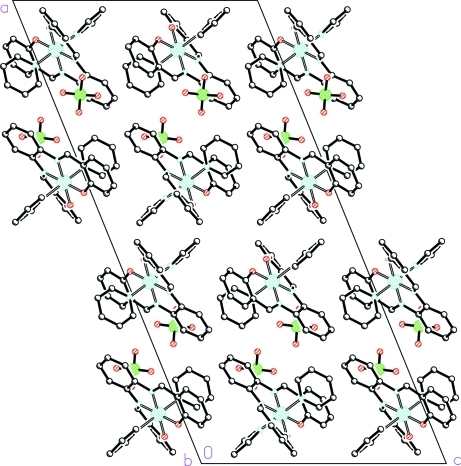
The crystal packing of (I), viewed along the b axis.

## supplementary crystallographic information

### Comment

Transition metal Schiff-base complexes with the tetradentate ligand configuration N_2_O_2_ have been extensively studied (Yamada *et al.*, 1999; Schenk *et al.*, 2007). Some of these complexes have interesting applications, *e.g.*, their ability to reversibly bind oxygen, and their use in catalysis for oxygenation and oxidation reactions of organic compounds (Nishinaga *et al.*, 1980; Park *et al.*, 1998; Speiser *et al.*, 1995). Among these metal complexes, cobalt(III) Schiff base complexes with two amines in axial positions have especially attracted interest due to their ability as antimicrobial agents (Kumar *et al.*, 2009; Miodragović *et al.*, 2006; Mishra *et al.*, 2008). The synthesis and reactivity of these complexes have also been playing an important part in the development of coordination chemistry. In this context, we herein report the synthesis and structure of the title compound, [Co(salophen)(py)_2_]ClO_4_, (I), and make a brief comparison with reported structures.

As illustrated in Fig. 1, compound (I) is the perchlorate salt of a mononuclear cobalt(III) complex cation. Bond distances and angles are given in the supplementary materials. The Schiff base ligand acts as a tetradentate ligand. The coordination polyhedron about Co is approximately octahedral, with a point group symmetry close to *Cs.* The three *trans* angles at the Co(III) centre are close to 180° and all other angles are close to 90°, ranging from 84.41 (5)° to 95.86 (5)°. The Co–O and Co–N distances of the coordinated salophen in the equatorial plane, Co(1)–O(1) = 1.8833 (10) Å, Co(1)–O(2) = 1.8806 (10) Å, Co(1)–N(1) = 1.8947 (12) Å, Co(1)–N(2) = 1.8953 (12) Å, are comparable with the Co–O and Co–N distances found in the related complexes [Co^III^(salophen)(morpholine)_2_]ClO_4 _and [Co^III^(salophen)(pyrrolidine)_2_]ClO_4_, [Co–O_av_ = 1.8815 (2) Å, Co–N_av_ = 1.8925 (2) Å, Amirnasr *et al.*, 2001], and [Co^III^(salophen)(4-picoline)_2_]ClO_4_.CH_2_Cl_2_ [Co–O_av_ = 1.888 (3) Å, Co–N_av_ = 1.906 (4) Å, Khandar *et al.*, 2007]. The salophen ligands in the title compound and in [Co^III^(salophen)(4-picoline)_2_]ClO_4_.CH_2_Cl_2_ [Khandar *et al.*, 2007] share a similar strong distortion, each having one phenolate moiety distinctly bent off from the least-squares plane of the remaining salophen ligand atoms, as is schematically shown by the chemical diagram of (I) and by the fact that in (I) the angle between the two least squares planes of phenolate O(1)—C(1)—C(2)—C(3)—C(4)—C(5)—C(6) and the rest of the salophen ligand is 25.27 (6)°. The two complexes differ however in the mutual orientations of the pyridine/picoline ligands – nearly perpendicular in (I) (interplanar angle 86.11 (5)°), but almost parallel in the picoline compound.

In the crystal structure of (I), the Co complexes are stacked in layers parallel to (100) with four layers per unit cell and Co at *x* = 0.107, 0.393, 0.607, and 0.823 (Fig. 2). Coherence of the structure is provided by a variety of C—H···O interactions (Table 1; contains only interactions with C—H···O angles > 110°) and by π-π stacking between symmetry equivalent pairs of pyridine rings N(4) through C(30) (centroid–centroid distance 3.652 (1) Å, ring–ring dihedral angle 10.5°, ring slippage 0.51 Å, shortest interatomic distances C30–C30(-*x*,*y*,1/2 - *z*) = 3.428 (3) Å and N4–C29(-*x*,*y*,1/2 - *z*) = 3.534 (2) Å).

### Experimental

To a stirring solution of Co(CH_3_COO)_2_.4H_2_O (0.125 g, 0.5 mmol) in methanol (25 ml) was added an equimolar of salophen (0.158 g, 0.5 mmol). The red solution turned brown immediately upon the formation of [Co^II^(salophen)] complex. To this solution was added 4 mmol of pyridine, and air was bubbled through the reaction mixture for about 3 h. To the resulting brown solution was then added 0.5 mmol (0.0615 g) of NaClO_4_and stirred for 5 minutes. Brown crystals of the complex suitable for X-ray crystallography were obtained after three days by slow evaporation of the methanol. The crystals were filtered off and washed with a small amount of cold methanol and dried under vacuum. Yield: 80%.

### Refinement

All H atoms attached to C atoms were fixed geometrically and treated as riding with C—H = 0.95Å and *U*_iso_(H) = 1.2 *U*_eq_.

### Crystal data

**Table:**

[Co(C_20_H_14_N_2_O_2_)(C_5_H_5_N)_2_]ClO_4_	*F*(000) = 2592
*M**_r_* = 630.91	*D*_x_ = 1.555 Mg m^−^^3^
Monoclinic, *C*2/*c*	Mo *K*α radiation, λ = 0.71073 Å
Hall symbol: -C 2yc	Cell parameters from 9429 reflections
*a* = 33.4032 (16) Å	θ = 2.3–30.1°
*b* = 10.6586 (5) Å	µ = 0.79 mm^−^^1^
*c* = 16.3498 (8) Å	*T* = 200 K
β = 112.179 (1)°	Prism, brown
*V* = 5390.3 (4) Å^3^	0.44 × 0.18 × 0.07 mm
*Z* = 8	

### Data collection

**Table:**

Bruker APEXII CCD diffractometer	7804 independent reflections
Radiation source: fine-focus sealed tube	6268 reflections with *I* > 2σ(*I*)
graphite	*R*_int_ = 0.024
φ and ω scans	θ_max_ = 30.0°, θ_min_ = 2.5°
Absorption correction: multi-scan (*SADABS*; Bruker, 2008)	*h* = −46→31
*T*_min_ = 0.86, *T*_max_ = 0.95	*k* = −14→14
24638 measured reflections	*l* = −22→22

### Refinement

**Table:**

Refinement on *F*^2^	Primary atom site location: structure-invariant direct methods
Least-squares matrix: full	Secondary atom site location: difference Fourier map
*R*[*F*^2^ > 2σ(*F*^2^)] = 0.031	Hydrogen site location: inferred from neighbouring sites
*wR*(*F*^2^) = 0.086	H-atom parameters constrained
*S* = 1.02	*w* = 1/[σ^2^(*F*_o_^2^) + (0.0413*P*)^2^ + 3.8856*P*] where *P* = (*F*_o_^2^ + 2*F*_c_^2^)/3
7804 reflections	(Δ/σ)_max_ = 0.001
379 parameters	Δρ_max_ = 0.38 e Å^−^^3^
0 restraints	Δρ_min_ = −0.40 e Å^−^^3^

### Special details

**Table:**

Experimental. Thin prisms from methanol. Bruker Kappa *APEX*II CCD diffractometer, full-sphere data collection. The temperature of 200 K was selected because crystals cracked at 100 K.
Geometry. All e.s.d.'s (except the e.s.d. in the dihedral angle between two l.s. planes) are estimated using the full covariance matrix. The cell e.s.d.'s are taken into account individually in the estimation of e.s.d.'s in distances, angles and torsion angles; correlations between e.s.d.'s in cell parameters are only used when they are defined by crystal symmetry. An approximate (isotropic) treatment of cell e.s.d.'s is used for estimating e.s.d.'s involving l.s. planes.
Refinement. Refinement of *F*^2^ against ALL reflections. The weighted *R*-factor *wR* and goodness of fit *S* are based on *F*^2^, conventional *R*-factors *R* are based on *F*, with *F* set to zero for negative *F*^2^. The threshold expression of *F*^2^ > σ(*F*^2^) is used only for calculating *R*-factors(gt) *etc*. and is not relevant to the choice of reflections for refinement. *R*-factors based on *F*^2^ are statistically about twice as large as those based on *F*, and *R*- factors based on ALL data will be even larger.

### Fractional atomic coordinates and isotropic or equivalent isotropic displacement parameters (Å^2^)

**Table:**

	*x*	*y*	*z*	*U*_iso_*/*U*_eq_	
Co1	0.107115 (6)	0.532208 (17)	0.407335 (12)	0.02150 (6)	
O1	0.05857 (3)	0.63179 (9)	0.39475 (7)	0.0278 (2)	
O2	0.08497 (3)	0.42434 (10)	0.47107 (7)	0.0271 (2)	
N1	0.12843 (4)	0.64456 (11)	0.34353 (8)	0.0235 (2)	
N2	0.15559 (4)	0.43132 (11)	0.41820 (8)	0.0226 (2)	
N3	0.14061 (4)	0.62115 (11)	0.51660 (8)	0.0253 (2)	
N4	0.07267 (4)	0.44507 (11)	0.29606 (8)	0.0244 (2)	
C1	0.05714 (5)	0.75357 (13)	0.38370 (10)	0.0264 (3)	
C2	0.02490 (5)	0.82090 (15)	0.40153 (12)	0.0369 (4)	
H2	0.0047	0.7764	0.4189	0.044*	
C3	0.02208 (6)	0.94902 (16)	0.39429 (14)	0.0433 (4)	
H3	0.0003	0.9918	0.4073	0.052*	
C4	0.05072 (6)	1.01725 (16)	0.36813 (14)	0.0437 (4)	
H4	0.0489	1.1061	0.3644	0.052*	
C5	0.08176 (6)	0.95457 (15)	0.34766 (12)	0.0369 (3)	
H5	0.1010	1.0010	0.3287	0.044*	
C6	0.08562 (5)	0.82224 (13)	0.35431 (10)	0.0276 (3)	
C7	0.11850 (5)	0.76275 (13)	0.33164 (9)	0.0265 (3)	
H7	0.1341	0.8133	0.3061	0.032*	
C8	0.16154 (4)	0.58912 (13)	0.32072 (9)	0.0243 (2)	
C9	0.17806 (5)	0.63868 (15)	0.26110 (10)	0.0294 (3)	
H9	0.1667	0.7144	0.2305	0.035*	
C10	0.21117 (5)	0.57614 (16)	0.24715 (10)	0.0318 (3)	
H10	0.2222	0.6081	0.2056	0.038*	
C11	0.22845 (5)	0.46652 (15)	0.29348 (11)	0.0316 (3)	
H11	0.2520	0.4264	0.2849	0.038*	
C12	0.21183 (5)	0.41546 (15)	0.35168 (10)	0.0289 (3)	
H12	0.2236	0.3403	0.3827	0.035*	
C13	0.17757 (4)	0.47570 (13)	0.36432 (9)	0.0234 (2)	
C14	0.16751 (5)	0.33115 (13)	0.46680 (9)	0.0251 (3)	
H14	0.1938	0.2922	0.4708	0.030*	
C15	0.14396 (5)	0.27522 (13)	0.51476 (9)	0.0263 (3)	
C16	0.16013 (6)	0.16128 (15)	0.55987 (10)	0.0339 (3)	
H16	0.1865	0.1283	0.5599	0.041*	
C17	0.13848 (6)	0.09751 (16)	0.60357 (11)	0.0399 (4)	
H17	0.1496	0.0208	0.6329	0.048*	
C18	0.09986 (6)	0.14678 (16)	0.60434 (11)	0.0375 (3)	
H18	0.0848	0.1031	0.6346	0.045*	
C19	0.08332 (5)	0.25753 (15)	0.56190 (10)	0.0320 (3)	
H19	0.0574	0.2902	0.5647	0.038*	
C20	0.10420 (5)	0.32399 (13)	0.51409 (9)	0.0252 (3)	
C21	0.12183 (5)	0.64920 (15)	0.57425 (10)	0.0321 (3)	
H21	0.0938	0.6176	0.5640	0.039*	
C22	0.14240 (6)	0.72265 (17)	0.64774 (11)	0.0385 (4)	
H22	0.1286	0.7408	0.6876	0.046*	
C23	0.18298 (6)	0.76947 (16)	0.66309 (11)	0.0373 (3)	
H23	0.1972	0.8217	0.7127	0.045*	
C24	0.20263 (5)	0.73919 (16)	0.60521 (11)	0.0352 (3)	
H24	0.2308	0.7693	0.6147	0.042*	
C25	0.18075 (5)	0.66448 (15)	0.53339 (10)	0.0299 (3)	
H25	0.1946	0.6427	0.4942	0.036*	
C26	0.07423 (5)	0.31985 (14)	0.28919 (10)	0.0301 (3)	
H26	0.0932	0.2735	0.3381	0.036*	
C27	0.04929 (6)	0.25592 (16)	0.21358 (11)	0.0377 (4)	
H27	0.0513	0.1672	0.2106	0.045*	
C28	0.02138 (5)	0.32254 (17)	0.14223 (11)	0.0373 (3)	
H28	0.0036	0.2805	0.0899	0.045*	
C29	0.01988 (5)	0.45122 (17)	0.14867 (11)	0.0357 (3)	
H29	0.0013	0.4994	0.1004	0.043*	
C30	0.04581 (5)	0.50935 (15)	0.22635 (10)	0.0309 (3)	
H30	0.0445	0.5981	0.2305	0.037*	
Cl1	0.203707 (13)	0.02747 (4)	0.38080 (2)	0.03354 (9)	
O3	0.24141 (5)	0.10462 (14)	0.41527 (11)	0.0619 (4)	
O4	0.16645 (5)	0.10872 (14)	0.34374 (11)	0.0570 (4)	
O5	0.19906 (7)	−0.04830 (17)	0.44767 (11)	0.0740 (5)	
O6	0.20593 (6)	−0.04926 (15)	0.31171 (11)	0.0634 (4)	

### Atomic displacement parameters (Å^2^)

**Table:**

	*U*^11^	*U*^22^	*U*^33^	*U*^12^	*U*^13^	*U*^23^
Co1	0.01951 (9)	0.02155 (9)	0.02323 (10)	−0.00096 (7)	0.00782 (7)	0.00160 (7)
O1	0.0234 (5)	0.0236 (4)	0.0379 (6)	0.0002 (4)	0.0133 (4)	0.0029 (4)
O2	0.0244 (5)	0.0270 (5)	0.0314 (5)	−0.0001 (4)	0.0122 (4)	0.0058 (4)
N1	0.0224 (5)	0.0250 (5)	0.0231 (5)	−0.0015 (4)	0.0086 (4)	0.0012 (4)
N2	0.0202 (5)	0.0240 (5)	0.0222 (5)	−0.0011 (4)	0.0066 (4)	−0.0006 (4)
N3	0.0261 (5)	0.0261 (5)	0.0240 (5)	−0.0002 (4)	0.0097 (4)	0.0004 (4)
N4	0.0198 (5)	0.0269 (5)	0.0251 (5)	−0.0012 (4)	0.0069 (4)	0.0010 (4)
C1	0.0236 (6)	0.0248 (6)	0.0294 (7)	0.0008 (5)	0.0085 (5)	0.0021 (5)
C2	0.0328 (8)	0.0305 (7)	0.0540 (10)	0.0039 (6)	0.0240 (7)	0.0056 (7)
C3	0.0427 (9)	0.0335 (8)	0.0627 (12)	0.0093 (7)	0.0300 (9)	0.0058 (8)
C4	0.0489 (10)	0.0247 (7)	0.0651 (12)	0.0060 (7)	0.0299 (9)	0.0058 (7)
C5	0.0384 (8)	0.0264 (6)	0.0504 (10)	0.0007 (6)	0.0219 (7)	0.0059 (7)
C6	0.0273 (7)	0.0244 (6)	0.0310 (7)	0.0007 (5)	0.0110 (5)	0.0036 (5)
C7	0.0272 (7)	0.0257 (6)	0.0267 (7)	−0.0022 (5)	0.0104 (5)	0.0034 (5)
C8	0.0217 (6)	0.0273 (6)	0.0236 (6)	−0.0029 (5)	0.0082 (5)	−0.0019 (5)
C9	0.0285 (7)	0.0333 (7)	0.0276 (7)	−0.0027 (5)	0.0121 (6)	0.0018 (6)
C10	0.0290 (7)	0.0414 (8)	0.0280 (7)	−0.0057 (6)	0.0142 (6)	−0.0028 (6)
C11	0.0261 (7)	0.0383 (8)	0.0328 (8)	−0.0014 (6)	0.0140 (6)	−0.0063 (6)
C12	0.0252 (6)	0.0312 (7)	0.0303 (7)	0.0006 (5)	0.0108 (5)	−0.0026 (6)
C13	0.0203 (6)	0.0269 (6)	0.0222 (6)	−0.0030 (5)	0.0070 (5)	−0.0035 (5)
C14	0.0227 (6)	0.0260 (6)	0.0228 (6)	0.0014 (5)	0.0042 (5)	−0.0005 (5)
C15	0.0281 (6)	0.0265 (6)	0.0213 (6)	−0.0001 (5)	0.0061 (5)	0.0012 (5)
C16	0.0394 (8)	0.0308 (7)	0.0278 (7)	0.0051 (6)	0.0083 (6)	0.0039 (6)
C17	0.0529 (10)	0.0299 (7)	0.0326 (8)	0.0018 (7)	0.0113 (7)	0.0095 (6)
C18	0.0451 (9)	0.0355 (8)	0.0298 (8)	−0.0089 (7)	0.0115 (7)	0.0069 (6)
C19	0.0314 (7)	0.0354 (7)	0.0281 (7)	−0.0051 (6)	0.0099 (6)	0.0052 (6)
C20	0.0264 (6)	0.0253 (6)	0.0212 (6)	−0.0042 (5)	0.0059 (5)	0.0011 (5)
C21	0.0325 (7)	0.0372 (8)	0.0300 (7)	0.0013 (6)	0.0158 (6)	0.0001 (6)
C22	0.0444 (9)	0.0448 (9)	0.0294 (8)	0.0056 (7)	0.0175 (7)	−0.0039 (6)
C23	0.0449 (9)	0.0350 (8)	0.0270 (7)	0.0012 (7)	0.0077 (6)	−0.0062 (6)
C24	0.0339 (8)	0.0372 (8)	0.0311 (7)	−0.0070 (6)	0.0084 (6)	−0.0053 (6)
C25	0.0283 (7)	0.0342 (7)	0.0275 (7)	−0.0040 (6)	0.0109 (6)	−0.0039 (6)
C26	0.0300 (7)	0.0273 (6)	0.0284 (7)	−0.0033 (6)	0.0059 (6)	0.0014 (5)
C27	0.0413 (9)	0.0313 (7)	0.0353 (8)	−0.0063 (6)	0.0086 (7)	−0.0048 (6)
C28	0.0306 (8)	0.0468 (8)	0.0287 (7)	−0.0050 (7)	0.0045 (6)	−0.0070 (6)
C29	0.0260 (7)	0.0476 (8)	0.0278 (7)	0.0061 (6)	0.0037 (6)	0.0016 (6)
C30	0.0271 (7)	0.0321 (7)	0.0302 (7)	0.0055 (6)	0.0069 (5)	0.0022 (5)
Cl1	0.0386 (2)	0.03535 (19)	0.02889 (18)	−0.00695 (15)	0.01530 (15)	−0.00310 (14)
O3	0.0448 (7)	0.0543 (8)	0.0685 (10)	−0.0165 (6)	0.0010 (7)	−0.0043 (7)
O4	0.0409 (7)	0.0565 (8)	0.0734 (10)	0.0025 (6)	0.0215 (7)	0.0056 (7)
O5	0.1066 (14)	0.0717 (11)	0.0531 (9)	−0.0091 (9)	0.0409 (10)	0.0203 (8)
O6	0.0824 (11)	0.0645 (9)	0.0529 (9)	−0.0057 (8)	0.0366 (8)	−0.0224 (7)

### Geometric parameters (Å, °)

**Table:**

Co1—O2	1.8806 (10)	C12—C13	1.394 (2)
Co1—O1	1.8833 (10)	C12—H12	0.9500
Co1—N1	1.8947 (12)	C14—C15	1.433 (2)
Co1—N2	1.8953 (12)	C14—H14	0.9500
Co1—N3	1.9577 (12)	C15—C16	1.418 (2)
Co1—N4	1.9789 (12)	C15—C20	1.422 (2)
O1—C1	1.3089 (17)	C16—C17	1.373 (2)
O2—C20	1.3078 (17)	C16—H16	0.9500
N1—C7	1.2985 (18)	C17—C18	1.397 (3)
N1—C8	1.4222 (18)	C17—H17	0.9500
N2—C14	1.3004 (18)	C18—C19	1.375 (2)
N2—C13	1.4238 (18)	C18—H18	0.9500
N3—C25	1.3448 (19)	C19—C20	1.418 (2)
N3—C21	1.3484 (19)	C19—H19	0.9500
N4—C26	1.3420 (19)	C21—C22	1.380 (2)
N4—C30	1.3421 (19)	C21—H21	0.9500
C1—C2	1.413 (2)	C22—C23	1.376 (3)
C1—C6	1.420 (2)	C22—H22	0.9500
C2—C3	1.371 (2)	C23—C24	1.378 (2)
C2—H2	0.9500	C23—H23	0.9500
C3—C4	1.391 (3)	C24—C25	1.378 (2)
C3—H3	0.9500	C24—H24	0.9500
C4—C5	1.377 (2)	C25—H25	0.9500
C4—H4	0.9500	C26—C27	1.382 (2)
C5—C6	1.417 (2)	C26—H26	0.9500
C5—H5	0.9500	C27—C28	1.383 (2)
C6—C7	1.433 (2)	C27—H27	0.9500
C7—H7	0.9500	C28—C29	1.378 (3)
C8—C9	1.393 (2)	C28—H28	0.9500
C8—C13	1.403 (2)	C29—C30	1.384 (2)
C9—C10	1.382 (2)	C29—H29	0.9500
C9—H9	0.9500	C30—H30	0.9500
C10—C11	1.394 (2)	Cl1—O5	1.4142 (15)
C10—H10	0.9500	Cl1—O6	1.4190 (14)
C11—C12	1.382 (2)	Cl1—O3	1.4296 (14)
C11—H11	0.9500	Cl1—O4	1.4479 (15)
			
O2—Co1—O1	84.41 (4)	C11—C12—H12	120.4
O2—Co1—N1	178.39 (5)	C13—C12—H12	120.4
O1—Co1—N1	94.06 (5)	C12—C13—C8	119.92 (13)
O2—Co1—N2	95.85 (5)	C12—C13—N2	125.63 (13)
O1—Co1—N2	179.15 (5)	C8—C13—N2	114.44 (12)
N1—Co1—N2	85.69 (5)	N2—C14—C15	124.82 (13)
O2—Co1—N3	89.96 (5)	N2—C14—H14	117.6
O1—Co1—N3	89.93 (5)	C15—C14—H14	117.6
N1—Co1—N3	89.55 (5)	C16—C15—C20	119.23 (14)
N2—Co1—N3	90.89 (5)	C16—C15—C14	117.44 (14)
O2—Co1—N4	90.50 (5)	C20—C15—C14	123.19 (13)
O1—Co1—N4	89.22 (5)	C17—C16—C15	121.51 (16)
N1—Co1—N4	89.96 (5)	C17—C16—H16	119.2
N2—Co1—N4	89.97 (5)	C15—C16—H16	119.2
N3—Co1—N4	178.98 (5)	C16—C17—C18	119.21 (15)
C1—O1—Co1	123.99 (9)	C16—C17—H17	120.4
C20—O2—Co1	125.60 (9)	C18—C17—H17	120.4
C7—N1—C8	122.95 (12)	C19—C18—C17	120.97 (15)
C7—N1—Co1	124.59 (10)	C19—C18—H18	119.5
C8—N1—Co1	111.89 (9)	C17—C18—H18	119.5
C14—N2—C13	122.71 (12)	C18—C19—C20	121.28 (16)
C14—N2—Co1	125.23 (10)	C18—C19—H19	119.4
C13—N2—Co1	112.04 (9)	C20—C19—H19	119.4
C25—N3—C21	118.18 (13)	O2—C20—C19	117.45 (13)
C25—N3—Co1	122.34 (10)	O2—C20—C15	124.79 (13)
C21—N3—Co1	119.26 (10)	C19—C20—C15	117.74 (13)
C26—N4—C30	118.12 (13)	N3—C21—C22	121.62 (15)
C26—N4—Co1	121.08 (10)	N3—C21—H21	119.2
C30—N4—Co1	120.76 (10)	C22—C21—H21	119.2
O1—C1—C2	117.78 (13)	C23—C22—C21	119.79 (16)
O1—C1—C6	124.30 (13)	C23—C22—H22	120.1
C2—C1—C6	117.91 (13)	C21—C22—H22	120.1
C3—C2—C1	121.39 (15)	C22—C23—C24	118.79 (15)
C3—C2—H2	119.3	C22—C23—H23	120.6
C1—C2—H2	119.3	C24—C23—H23	120.6
C2—C3—C4	120.97 (16)	C25—C24—C23	118.93 (16)
C2—C3—H3	119.5	C25—C24—H24	120.5
C4—C3—H3	119.5	C23—C24—H24	120.5
C5—C4—C3	119.30 (15)	N3—C25—C24	122.65 (15)
C5—C4—H4	120.3	N3—C25—H25	118.7
C3—C4—H4	120.3	C24—C25—H25	118.7
C4—C5—C6	121.29 (15)	N4—C26—C27	122.41 (14)
C4—C5—H5	119.4	N4—C26—H26	118.8
C6—C5—H5	119.4	C27—C26—H26	118.8
C5—C6—C1	119.08 (14)	C26—C27—C28	119.22 (16)
C5—C6—C7	118.49 (14)	C26—C27—H27	120.4
C1—C6—C7	122.43 (13)	C28—C27—H27	120.4
N1—C7—C6	124.61 (13)	C29—C28—C27	118.61 (15)
N1—C7—H7	117.7	C29—C28—H28	120.7
C6—C7—H7	117.7	C27—C28—H28	120.7
C9—C8—C13	120.43 (13)	C28—C29—C30	119.17 (15)
C9—C8—N1	125.36 (13)	C28—C29—H29	120.4
C13—C8—N1	114.21 (12)	C30—C29—H29	120.4
C10—C9—C8	119.06 (14)	N4—C30—C29	122.47 (15)
C10—C9—H9	120.5	N4—C30—H30	118.8
C8—C9—H9	120.5	C29—C30—H30	118.8
C9—C10—C11	120.52 (14)	O5—Cl1—O6	109.89 (11)
C9—C10—H10	119.7	O5—Cl1—O3	111.16 (11)
C11—C10—H10	119.7	O6—Cl1—O3	110.03 (11)
C12—C11—C10	120.86 (14)	O5—Cl1—O4	109.71 (11)
C12—C11—H11	119.6	O6—Cl1—O4	107.85 (10)
C10—C11—H11	119.6	O3—Cl1—O4	108.12 (9)
C11—C12—C13	119.11 (14)		
			
O2—Co1—O1—C1	153.07 (12)	C5—C6—C7—N1	172.43 (15)
N1—Co1—O1—C1	−26.44 (12)	C1—C6—C7—N1	−7.1 (2)
N3—Co1—O1—C1	63.11 (12)	C7—N1—C8—C9	−20.5 (2)
N4—Co1—O1—C1	−116.34 (12)	Co1—N1—C8—C9	167.85 (12)
O1—Co1—O2—C20	−173.59 (12)	C7—N1—C8—C13	159.31 (13)
N2—Co1—O2—C20	7.22 (12)	Co1—N1—C8—C13	−12.36 (14)
N3—Co1—O2—C20	−83.66 (12)	C13—C8—C9—C10	−1.6 (2)
N4—Co1—O2—C20	97.24 (12)	N1—C8—C9—C10	178.21 (13)
O1—Co1—N1—C7	21.49 (12)	C8—C9—C10—C11	−1.4 (2)
N2—Co1—N1—C7	−159.33 (12)	C9—C10—C11—C12	2.5 (2)
N3—Co1—N1—C7	−68.41 (12)	C10—C11—C12—C13	−0.6 (2)
N4—Co1—N1—C7	110.70 (12)	C11—C12—C13—C8	−2.4 (2)
O1—Co1—N1—C8	−167.01 (9)	C11—C12—C13—N2	176.48 (13)
N2—Co1—N1—C8	12.17 (9)	C9—C8—C13—C12	3.5 (2)
N3—Co1—N1—C8	103.10 (9)	N1—C8—C13—C12	−176.31 (12)
N4—Co1—N1—C8	−77.80 (9)	C9—C8—C13—N2	−175.50 (12)
O2—Co1—N2—C14	−7.55 (12)	N1—C8—C13—N2	4.70 (17)
N1—Co1—N2—C14	171.98 (12)	C14—N2—C13—C12	4.6 (2)
N3—Co1—N2—C14	82.50 (12)	Co1—N2—C13—C12	−173.76 (11)
N4—Co1—N2—C14	−98.05 (12)	C14—N2—C13—C8	−176.42 (12)
O2—Co1—N2—C13	170.81 (9)	Co1—N2—C13—C8	5.17 (14)
N1—Co1—N2—C13	−9.66 (9)	C13—N2—C14—C15	−173.08 (13)
N3—Co1—N2—C13	−99.15 (9)	Co1—N2—C14—C15	5.1 (2)
N4—Co1—N2—C13	80.30 (9)	N2—C14—C15—C16	175.93 (13)
O2—Co1—N3—C25	142.20 (12)	N2—C14—C15—C20	0.2 (2)
O1—Co1—N3—C25	−133.39 (12)	C20—C15—C16—C17	−0.6 (2)
N1—Co1—N3—C25	−39.34 (12)	C14—C15—C16—C17	−176.47 (15)
N2—Co1—N3—C25	46.35 (12)	C15—C16—C17—C18	−0.7 (3)
O2—Co1—N3—C21	−43.24 (12)	C16—C17—C18—C19	0.2 (3)
O1—Co1—N3—C21	41.17 (12)	C17—C18—C19—C20	1.6 (2)
N1—Co1—N3—C21	135.23 (12)	Co1—O2—C20—C19	177.06 (10)
N2—Co1—N3—C21	−139.09 (12)	Co1—O2—C20—C15	−4.4 (2)
O2—Co1—N4—C26	−51.73 (12)	C18—C19—C20—O2	175.84 (14)
O1—Co1—N4—C26	−136.13 (12)	C18—C19—C20—C15	−2.8 (2)
N1—Co1—N4—C26	129.81 (12)	C16—C15—C20—O2	−176.25 (14)
N2—Co1—N4—C26	44.12 (12)	C14—C15—C20—O2	−0.6 (2)
O2—Co1—N4—C30	125.70 (12)	C16—C15—C20—C19	2.3 (2)
O1—Co1—N4—C30	41.29 (12)	C14—C15—C20—C19	177.89 (13)
N1—Co1—N4—C30	−52.76 (12)	C25—N3—C21—C22	1.5 (2)
N2—Co1—N4—C30	−138.45 (12)	Co1—N3—C21—C22	−173.33 (12)
Co1—O1—C1—C2	−161.34 (12)	N3—C21—C22—C23	0.3 (3)
Co1—O1—C1—C6	19.2 (2)	C21—C22—C23—C24	−1.5 (3)
O1—C1—C2—C3	177.90 (17)	C22—C23—C24—C25	0.8 (3)
C6—C1—C2—C3	−2.6 (3)	C21—N3—C25—C24	−2.1 (2)
C1—C2—C3—C4	0.7 (3)	Co1—N3—C25—C24	172.49 (12)
C2—C3—C4—C5	1.2 (3)	C23—C24—C25—N3	1.0 (3)
C3—C4—C5—C6	−1.2 (3)	C30—N4—C26—C27	−0.1 (2)
C4—C5—C6—C1	−0.7 (3)	Co1—N4—C26—C27	177.38 (13)
C4—C5—C6—C7	179.73 (17)	N4—C26—C27—C28	−0.5 (3)
O1—C1—C6—C5	−177.95 (15)	C26—C27—C28—C29	0.9 (3)
C2—C1—C6—C5	2.6 (2)	C27—C28—C29—C30	−0.8 (3)
O1—C1—C6—C7	1.6 (2)	C26—N4—C30—C29	0.2 (2)
C2—C1—C6—C7	−177.91 (15)	Co1—N4—C30—C29	−177.29 (12)
C8—N1—C7—C6	−179.53 (13)	C28—C29—C30—N4	0.3 (3)
Co1—N1—C7—C6	−8.9 (2)		

### Hydrogen-bond geometry (Å, °)

**Table:**

*D*—H···*A*	*D*—H	H···*A*	*D*···*A*	*D*—H···*A*
C5—H5···O4^i^	0.95	2.40	3.293 (3)	157
C11—H11···O6^ii^	0.95	2.49	3.261 (3)	138
C12—H12···O3	0.95	2.59	3.501 (2)	161
C14—H14···O3^iii^	0.95	2.51	3.002 (2)	112
C29—H29···O1^iv^	0.95	2.47	3.111 (2)	124

Symmetry codes: (i) *x*, *y*+1, *z*; (ii) −*x*+1/2, *y*+1/2, −*z*+1/2; (iii) −*x*+1/2, −*y*+1/2, −*z*+1; (iv) −*x*, *y*, −*z*+1/2.
